# Anti-Inflammatory and Analgesic Activity of Total Flavone of *Cunninghamia lanceolata*

**DOI:** 10.3390/molecules17088842

**Published:** 2012-07-25

**Authors:** Hai-Liang Xin, Xiao-Feng Zhai, Xu Zheng, Lei Zhang, Yu-Liang Wang, Zhuo Wang

**Affiliations:** 1 Department of Traditional Chinese Medicine, Changhai Hospital, Second Military Medical University, Shanghai 200433, China; 2 Department of Naval Medicine, Second Military Medical University, Shanghai 200433, China; 3 Department of Pharmacognosy, School of Pharmacy, Second Military Medical University, Shanghai 200433, China; 4 Plant Biotechnology Research Center, School of Agriculture and Biology, Fudan-SJTU-Nottingham Plant Biotechnology R&D Center, Shanghai Jiao Tong University, Shanghai 200240, China; 5 Department of Pharmacy, Changhai Hospital, Second Military Medical University, Shanghai 200433, China

**Keywords:** *Cunninghamia lanceolata* (Lamb.) Hook, total flavone, anti-inflammatory, analgesia

## Abstract

The present study was undertaken to investigate the anti-inflammatory and analgesic activity of total flavone of branches and leaves of *Cunninghamia lanceolata* (TFC) to provide a scientific basis for its clinical use and resource development. TFC was evaluated for anti-inflammatory and analgesic activity in mice or rats using chemical and thermal models of nociception, including acetic acid-induced writhing test, hot plate latency test, formalin test and carrageenan induced paw oedema test. Results showed that TFC given orally can significantly attenuate acetic acid-induced writhing in mice in a dose-dependent manner. In the hot plate latency test, TFC showed common activity in prolonging duration time only at the highest dose (400 mg/kg). Each dose of TFC could not significantly inhibit the first phase but was active in the later phase of formalin-induced pain, whereas morphine showed notable activity in the two phases. In the carrageenan-induced paw oedema model, TFC could significantly and dose-dependently reduce the carrageenan-induced paw edema at the third and fifth hour, and decrease the content of PEG_2_ in paw edema tissue and that of COX-2 in blood serum. It may be concluded that TFC showed both anti-inflammatory and analgesic effects, showing that it can be of importance in drug development, especially in the field of pain and inflammation.

## 1. Introduction

*Cunninghamia lanceolata* (Lamb.) Hook., as an important native tree species that has been widely planted in mountainous areas in the tropics and subtropics in China for more than 1,000 years and has been used for a variety of wood products. Besides, in Chinese folklore medicine, the roots, branches, leaves, barks of *C. lanceolata* have been used orally to cure arthralgia, stomach ache, and winter cough, and externally used for fractures, burns, scalds and sensitization dermatitis [1]. As reported, most pharmacological studies have mainly focused on its antimicrobial action and some novel chemicals were isolated [2,3]. According to its traditional use, although *C. lanceolata* is effective in pain-relief as a folklore medicine, there still has no comprehensive evaluation for the anti-inflammatory and analgesic activity of this plant. Previously, we reported that several biflavones were isolated from the EtOH extract of branches and leaves of *C. lanceolata* [4,5]. In this present study, we will focus on investigating total flavone of branches and leaves of *C. lanceolata* (TFC) with mice nociception models induced by the chemical and thermal stimuli so as to elucidate its anti-inflammatory and analgesic activity and provide a scientific basis for the clinical use and resource development of *C. lanceolata*.

## 2. Results

**Figure 1:**
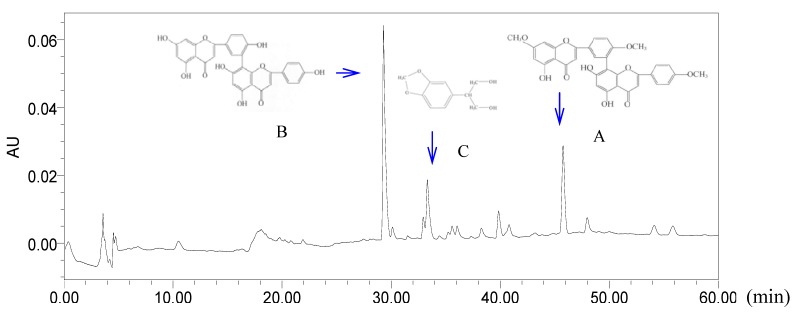
HPLC chromatogram of TFC (Major peaks were identified by comparison with standard compounds. A: sciadopitysin; B: amentoflavone; C: 2-(benzo[d][1,3]dioxol-5-yl)-propane-1,3-diol).

The HPLC analysis indicated that there were biflavones and other constituents in TFC and the major peaks, which were identified by comparing with standard compounds and literature [4], were sciadopitysin (A), amentoflavone (B) and 2-(benzo[d][1,3]dioxol-5-yl)-propane-1,3-diol (C), respectively (Figure 1).

Acute oral toxicity tests found the MTD of TFC for mice was >3,000 mg/kg, which indicated a good safety margin for TFC applications. In the acetic acid-induced writhing test, TFC could significantly and dose-dependently attenuate acetic acid induced writhing in mice (Table 1). In the hot plate latency test, the extract showed common activity, in prolonging the duration time only at the highest dose tested (400 mg/kg) (Table 1).

In the formalin test, each dose of the extract could not significantly inhibit the first phase, but was active in the later phase of formalin-induced pain, whereas morphine showed notable activity in both phases (Table 1). 

In the carrageenan induced paw oedema test, TFC could significantly and dose-dependently reduce the carrageenan-induced paw edema at the third and fifth hour (Table 2), and it could also decrease the content of PEG_2_ in paw edema tissue (Table 3) and that of COX-2 in blood serum (Table 4).

**Figure 2:**
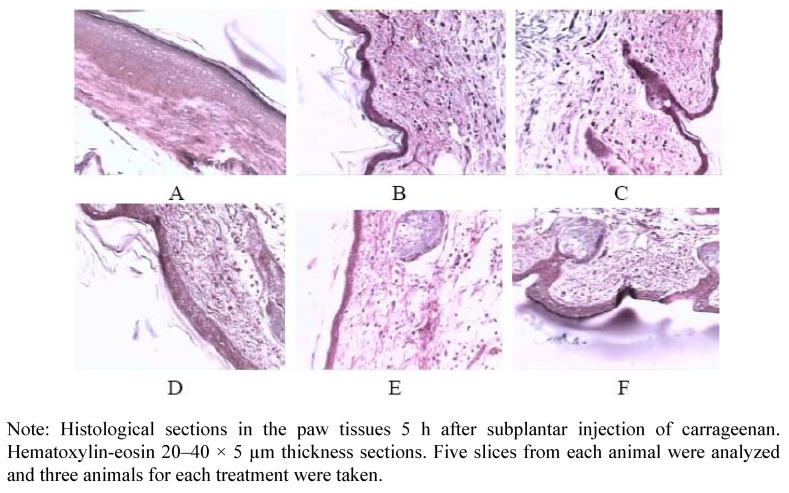
Effects of TFC administered orally on carrageenan-induced cellular infiltration. (**A**) normal tissue; (**B**) carrageenan+normal saline; (**C**) carrageenan+TFC (100 mg/kg); (**D**) carrageenan+TFC (200 mg/kg); (**E**) carrageenan+TFC (400 mg/kg); (**F**) carrageenan+ASA.

Histological assessment of sections from swollen feet of mice 5 h after carrageenan injection showed oedema and substantial infiltrates of acute inflammatory cells into the subplantar tissues, while the TFC administered group had lighter oedemas and fewer infiltrates of neutrophilic granulocyte (Figure 2). 

## 3. Discussion

Pain and inflammation are associated with the pathophysiology of various clinical conditions such as arthritis, cancer and vascular diseases. Inflammatory reactions are not only the response of living tissues to injury and infection, but also are relevant to disease development, such as in asthma, multiple sclerosis, colitis, inflammatory bowel disease and atherosclerosis. Screening of natural products used in traditional medical systems is one prospective way to relieve the symptoms of pain and inflammation [6]. 

It is known that non-steroidal anti-inflammatory drugs usually do not increase the pain threshold in normal tissues, whereas local anesthetics and narcotics do [7]. The acetic acid-induced writhing method is widely used for evaluation of peripheral anti-nociceptive activity [8]. The carrageenan-induced rat paw edema model, representing a form of acute inflammation, has been frequently used to assess the anti-inflammatory effects of natural products [9]. The hot plate test was undertaken to verify if TFC would have any central analgesic effect. In the acetic acid-induced writhing test, the data showed the extract produced a dose-dependent inhibition of writhing on nociception. In the hot plate test, the results for the group treated with TFC did not differ significantly from those obtained for the negative control group. On the other hand, the group treated with morphine showed a highly significant result. Hence, it is assumed that TFC has no analgesic effect on the central nervous system that would contribute to its peripheral analgesic effect. Standard NSAIDs like aspirin offer relief from inflammatory pain by suppressing the formation of pain causing substances in the peripheral tissues, where prostaglandin and bradykinin were suggested to play an important role in the pain process [10]. Therefore, TFC might may suppress the formation of these substances or antagonize the action of these substances to exert its peripheral analgesic activity.

The advantage of the formalin model of nociception is that it can discriminate pain in its central and/or peripheral components. The test consists of two different phases which can be separated in time: the first one is generated in the periphery through the activation of nociceptive neurons by the direct action of formalin and the second phase occurs through the activation of the ventral horn neurons at the spinal cord level. Central analgesic drugs, such as morphine, inhibited equally in both phases, whereas peripherally acting drugs, such as steroids (dexamethasone) and NSAIDs (aspirin) suppressed mainly pain in the later phase [11]. For the anti-inflammatory and analgesic activity of TFC in the formalin model this was also the case. From these investigations, it may be concluded that TFC showed both anti-inflammatory and analgesic effects. It is also suggested that the mechanism of action of TFC might be associated with the inhibition of prostaglandin synthesis, as observed for most non-steroidal drugs.

## 4. Experimental

### 4.1. Plant Material

Plant material was collected at Changshan County, Zhejiang Province in August 2007, and was identified by Prof. Han-Ming Zhang, School of Pharmacy, Second Military Medical University. A voucher specimen of *C. lanceolata* (No. 20070812) was deposited at the herbarium of Department of Traditional Chinese Medicine, Second Military Medical University.

### 4.2. Sample Preparation

The coarse powder of younger branches and leaves of *C. lanceolata* (10 kg) was percolated with 75% EtOH as solvent (200 L, 2 h, 26 °C). The concentrated extract was suspended in H_2_O and extracted with petroleum ether (60–90°), chloroform, acetoacetate and *n*-butanol (20 L), successively. The acetoacetate fraction was concentrated under reduced pressure to obtain TFC (92.8 g) for the activity tests.

### 4.3. Animal

Male wistar rats and ICR mice, weighing 180–200 g and 20–22 g (Shanghai SLAC Laboratory Animal Co., Ltd., Shanghai, China), respectively, were maintained on standard laboratory animal feed and water *ad libitum*. They were housed in polypropylene cages at room temperature with a 12 h light/dark cycle (6 am to 6 pm) and allowed to acclimatize with the laboratory environment for at least five days prior to the commencement of the study. These studies were carried out in accordance with the rules governing the use of laboratory animals as accepted internationally.

### 4.4. Drugs and Chemicals

Rat COX-2 ELISA kit was purchased from Shanghai Yanxin Biotech Co., Ltd., (Shanghai, China); EtOH, acetoacetate, chloroform, *n*-butanol, formalin and acetic acid (analytical grade) were purchased from Sinopharm Chemical Reagent Co. Ltd. (Shanghai, China). The morphine hydrochloride, acetylsalicylic acid (ASA) were manufactured by Qinghai Pharmaceutical Factory (Xining, China).

### 4.5. HPLC Fingerprint Analysis

HPLC fingerprint analysis was performed on a Waters 2695 HPLC system equipped with a quaternary pump, vacuum degasser, thermostatic column compartment and diode array detector (DAD). An Amethyst C18 column (4.6 × 250 mm, 5 μm) with an Extend C18 guard column (4.6 mm × 10 mm, 5 μm) was used. Gradient elution was employed using solvent systems A (methanol) and B (0.3% formic acid) at ambient temperature. The gradient program was used as follows: initial 0–12 min, A–B (15:85, v/v); 12–14 min, linear change to A–B (30:70, v/v); 14–20 min, A–B (30:70, v/v); 20–40 min, linear change to A–B (80:20, v/v); 40–45 min, linear change to A–B (90:10, v/v); 45–57 min, A–B (90:10, v/v); 57–59 min, linear change to A–B (15:85, v/v); 59–60 min, A–B (15:85, v/v). Flow rate was 1.0 mL/min and column temperature was maintained at 26 °C. The detector wavelength was 280 nm. An aliquot of 20 μL solution was injected for acquiring chromatograms.

### 4.6. Acute Toxicity Test

TFCs (3 g/10 mL of 5% Tween-80/kg body weight) were administered orally to mice (n = 10, each group). Then, animals were observed for any abnormal behavior for 3 h, and mortality was noted for up to 2 weeks. A group of animals treated with the Tween-80 served as the control.

### 4.7. Acetic Acid-Induced Writhing Test *[12]*

The mice were divided into five groups, each containing 10 mice. The mice in groups of vehicle control, positive control and test were treated with normal saline, acetylsalicylic acid and TFC, respectively. After 30 min of drug administration, the mice were treated with 0.6% acetic acid. The number of abdominal contractions of mice was counted for a period of 10 min after 5 min latency. 

### 4.8. Hot Plate Latency Test *[13]*

The hot plate latency test was carried out on groups of female mice as reported previously [7]. The animals were divided into five groups, each containing 10. Each of test animals was placed on a beaker maintained at 55 °C, 30 min after administration with normal saline, TFC or morphine. The cut-off time was 60 s in the hot-plate test in order to minimize skin damages.

### 4.9. Carrageenan Induced Paw Oedema Test *[14,15]*

The animals were divided into five groups, each containing six rats. Acute inflammation was induced by injecting 0.1 mL of (1%) carrageenan into the plantar surface of the rat hind paw. Each group was treated with TFC, normal saline or ASA as reference agent administered 30 min before carrageenan injection, and was treated as follows.

#### 4.9.1. Edema Inhibitory Activity

The paw size of the animals in each group was measured hourly for 5 h using cotton thread and a meter rule as previously described. Edema inhibitory activity of TFC was determined using the following expression:

$$\mathrm{Percentage}\;\mathrm{inhibition}(\%)=\frac{{{(C}_{1}{-C}_{0})}_{\mathrm{control}}{-(C}_{t}{-C}_{0}{)}_{\mathrm{treared}}}{{{(C}_{1}{-C}_{0})}_{\mathrm{control}}}\times 100\%$$

where C_t_, = paw circumference at time t, C_0_ = paw circumference before carrageenan injection, and C_t_ − C_0_ = edema formed.

#### 4.9.2. Content of COX-2 in Blood Serum

For determining content of COX-2 in blood serum, all rats were bled from eyes, and the blood was put in Eppendorfs tube and centrifuged for 10 min at 3000 r/min, then the supernatant was collected to determine the content of COX-2 according to the ELISA Kits instruction. 

#### 4.9.3. Content of PEG_2_ in Paw Edema Tissue

For determining content of PEG_2_ in paw edema tissue, carrageenan-induced feet were sheared from ankle joint, weighted, lacerated at *digiti pedis* position, and soaked in a test tube containing 2.5 mL of physiological saline with continuous shaking. The soaking solution was centrifuged for 15 min at 3,000 r/min, then 2 mL of KOH methanol solution (0.5 mol/L) was added into 0.15 mL of the supernatant. After 20 min incubation at 50 °C, 2.5 mL of methanol was added into it and was measured for OD value at 278 nm.

#### 4.9.4. Histological Assessment

For histological assessment, carrageenan-induced and control feet were removed post mortem 5 h after subplantar injection and fixed in 10% formalin buffered saline. After decalcification, the samples were embedded in paraffin, sectioned and stained with haematoxylin and eosin.

### 4.10. Formalin Test *[16]*

The animals were divided into five groups each containing 10 mice, and were administered with either normal saline, TFC or morphine. Thirty minutes after this treatment, 20 μL of a freshly prepared 2.5% solution of formalin was injected subcutaneously under the plantar surface of the left hind paw of each mouse. The duration of paw licking(s) as an index of painful response was determined at 0–5 min (early phase, neurogenic) and 20–35 min (late phase, inflammatory) after formalin injection.

### 4.11. Statistical Analysis

The results were expressed as mean ± SEM, and ANOVA was used to analyze and compare data, followed by Dunnet’s test for multiple comparisons. *P* < 0.05 was considered significant in all experiments.

## 5. Conclusions

The results obtained from the analgesic and anti-inflammatory studies strongly support the traditional use of *C. lanceolata* for the treatment of pain and rheumatic conditions. The facts underlying the observations in this study may be attributed to the activity of one or more phyto-chemical agents. These phytochemical constituents may include biflavones according to our previous report. It has reported that flavonoids such as quercetin is effective in acute inflammation [17]. In conclusion, this study has established the anti-inflammatory and analgesic activities of braches and leaves of *C. lanceolata*, showing that it can be of importance in drug development, especially in the field of pain and inflammation.

## Figures and Tables

**Table 1:** Analgesic activity of TFC and reference drug in the different tests.

Groups	Acetic acid-induced writhing (number of writhings)	Hot plate latency test (duration on the plate)	Formalin test	
			Paw licking time in early phase (s)	Paw licking time in late phase (s)
Normal saline (10 mL/kg. o.p.)	37.41 ± 4.91	14.36 ± 5.10	134.5 ± 28.3	171.4 ± 24.8
TFC (100 mg/kg. o.p.)	29.66 ± 5.02 *	13.27 ± 4.80	117.6 ± 20.4	132.1 ± 37.5 *
TFC (200 mg/kg. o.p.)	24.24 ± 3.81 **	15.31 ± 6.46	121.4 ± 18.5	110.3 ± 26.7 **
TFC (400 mg/kg. o.p.)	18.35 ± 4.54 **	18.39 ± 3.02 *	108.5 ± 20.0 *	96.2 ± 23.1 **
ASA (100 mg/kg. o.p.)	13.27 ± 5.49 **	/	/	/
Morphine (5 mg/kg. s.c.)	/	36.40 ± 1.69 **	19.3 ± 3.2 **	10.4 ± 8.3 **

Note: Each group represents the mean ± SEM (n = 10). Asterisks indicated significant difference from control. * *p* < 0.05, ** *p* < 0.01 (ANOVA followed by Dunnett’s test).

**Table 2:** Effects of TFC on carrageenan-induced paw edema in rats.

Group	Increase in paw size (edema) (mm)		Inhibition (%)	
	3 h	5 h	3 h	5 h
Normal saline (10 mL/kg. o.p.)	6.72 ± 0.34	4.80 ± 0.22	/	/
TFC (100 mg/kg. o.p.)	5.10 ± 0.21 **	3.49 ± 0.37 **	33.47	44.63
TFC (200 mg/kg. o.p.)	4.67 ± 0.42 **	3.04 ± 0.19 **	41.43	58.39
TFC (400 mg/kg. o.p.)	3.61 ± 0.36 *	2.86 ± 0.24 **	73.27	62.75
ASA (10 mg/kg. o.p.)	3.06 ± 0.30 **	2.13 ± 0.16 **	62.45	87.92

Note: Each group represents the mean ± SEM (n = 6). Asterisks indicated significant difference from control. * *p* < 0.05, ** *p* < 0.01 (ANOVA followed by Dunnett’s test).

**Table 3:** Effects of TFC on content of PEG_2_ in paw edema tissue induced by carrageenan.

Group	Content (OD value/g)
Normal saline (10 mL/kg. o.p.)	0.94 ± 0.12
TFC (100 mg/kg. o.p.)	0.47 ± 0.16 *
TFC (200 mg/kg. o.p.)	0.31 ± 0.20 **
TFC (400 mg/kg. o.p.)	0.28 ± 0.11 **
ASA (10 mg/kg. o.p.)	0.21 ± 0.14 **

Note: Content of PEG_2_ was expressed as OD/g of tissue and each group represents the mean ± SEM (n = 3). Asterisks indicated significant difference from control. * *p* < 0.05, ** *p* < 0.01 (ANOVA followed by Dunnett’s test).

**Table 4:** Effects of TFC on content of COX-2 in blood serum.

Group	Content (pg/mL)
Normal saline (10 mL/kg. o.p.)	874.64 ± 142.51
TFC (100 mg/kg. o.p.)	521.70 ± 193.65 *
TFC (200 mg/kg. o.p.)	328.75 ± 123.27 **
TFC (400 mg/kg. o.p.)	275.91 ± 105.38 **
ASA (10 mg/kg. o.p.)	217.61 ± 149.64 **

Note: Each group represents the mean ± SEM (n = 3). Asterisks indicated significant difference from control. * *p* < 0.05, ** *p* < 0.01 (ANOVA followed by Dunnett’s test).
